# Association between Levels of IgA Antibodies to Tissue Transglutaminase and Gliadin-Related Nonapeptides in Dermatitis Herpetiformis

**DOI:** 10.1100/2012/363296

**Published:** 2012-04-01

**Authors:** Justyna Gornowicz-Porowska, Monika Bowszyc-Dmochowska, Agnieszka Seraszek-Jaros, Elżbieta Kaczmarek, Marian Dmochowski

**Affiliations:** ^1^Cutaneous Histopathology and Immunopathology Section, Department of Dermatology, Poznan University of Medical Sciences, 49 Przybyszewskiego Street, 60-355 Poznan, Poland; ^2^Department of Bioinformatics and Computational Biology, Poznan University of Medical Sciences, 79 Dabrowskiego Street, 60-529 Poznan, Poland

## Abstract

Dermatitis herpetiformis (DH) is an autoimmunity-driven inflammatory blistering dermatosis associated with a gluten-dependent enteropathy. Tissue transglutaminase (tTG) and nonapeptides of gliadin (npG) are considered in its pathomechanism/diagnostics. Here, the diagnostic accuracy of anti-tTG/anti-npG IgA ELISAs in Slavic DH patients with active skin rash was assessed through creating receiver operating characteristic (ROC) curves, determining cutoff values, and calculating correlations between levels of anti-tTG/anti-npG IgA in DH, IgA/neutrophil-mediated non-DH patients and healthy persons. Altogether, sera from 80 Slavic individuals were examined. There were negligible differences between cutoff points obtained by the ELISAs manufacturer and those in this study. There were statistically significant correlations between levels of anti-tTG/anti-npG IgA in both DH group and the group of IgA/neutrophil-mediated non-DH dermatoses. There was no such correlation in healthy controls. It seems that IgA autoantibodies to tTG and npG in the IgA/neutrophil-mediated DH are produced in the coordinated way implying their causal relationship.

## 1. Introduction

**Figure 1:**
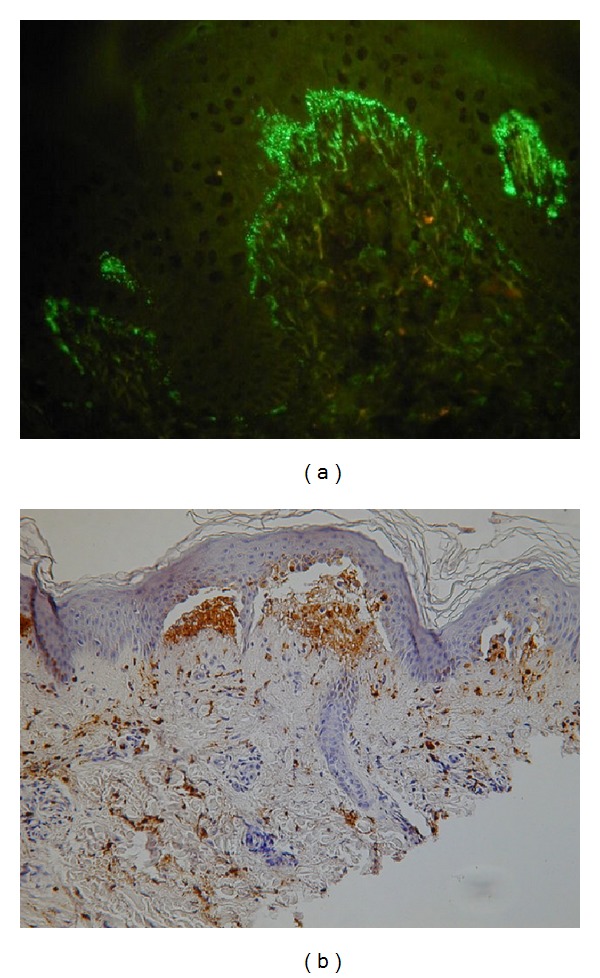
(a) Microgranular and fibrillar IgA1 deposits at dermal papillae in DIF in a young man with DH (original magnification ×400). (b) Neutrophil elastase deposits in immunohistochemistry in lesional skin in a middle-aged woman with DH (original magnification, ×200).

Dermatitis herpetiformis (DH) is a chronic, IgA-mediated, inflammatory dermatosis with intense itching and polymorphic eruption undergoing the spatial-temporal evolution [1]. In its pathomechanism, the role is played by both genetic and environmental factors. Thus, familial occurrence is sometimes observed [2]. It is universally thought that DH is associated with gluten-sensitive enteropathy (GSE), being a cutaneous manifestation of celiac disease (CD) [3]. These diseases are caused by an immune reaction to proline-rich gliadin, a prolamin (gluten protein) found in wheat [4]. However, the trigger/triggers of pathological antigliadin autoimmune response in DH and relationship between CD and DH still remain inadequately understood. Some studies indicated epidemiologic trends of increasing incidence of CD. DH is also an important medical issue demanding highly efficient medical and social services. DH is characterized by cutaneous microgranular IgA deposits in the dermal papillae (microgranular and fibrillar deposits are sometimes seen there) and/or along the dermal-epidermal junction [1]; however, interesting issue is which IgA subclass is dominant in cutaneous deposits. In humans, IgA1 is a predominant subclass in the sera, and IgA2 prevails in mucosal secretions of the colon [1]. Immunofluorescence analysis with monoclonal antibodies revealed that IgA1 without IgA2 was found in the cutaneous deposits in all four patients examined in an early study [5]. It was therefore speculated that both IgA1 and IgA2 may be produced in the pathologic gut-associated lymphoid tissue, but only IgA1 is involved in the production of cutaneous lesions [5]. Still, there are newer data that both IgA1 and IgA2 are forming IgA cutaneous deposits in DH, although IgA1 (Figure 1(a)) predominates [1, 6]. In the development of DH, important is the accumulation of activated (neutrophil elastase-secreting) neutrophils (Figure 1(b)) that are forming microabscesses in the dermal papillae with subsequent formation of microvesicles and finally subepidermal (intralamina lucida) blisters [7]. Main autoantigens in DH are enzymes of the transglutaminase family [8, 9]: epidermal transglutaminase (eTG) and closely related tissue transglutaminase (tTG). They are considered to be autoantigens plausibly recognized by principally IgA1 autoantibodies in this disease [10]. Recently, the role of nonapeptides of gliadin (npG) in pathomechanism of DH is considered [11]. Further, there are findings indicating that antibodies against deamidated synthetic gliadin-derived peptides are the most reliable tool in order to identify gluten sensitivity in DH patients [12]. Interestingly, recent data [13] indicated that cross-linking microbial TG (mTG) may reduce immunoreactivity of milk proteins. Cross-linking by mTG results in integration of milk proteins epitopes into newly created protein conglomerates, in such a way that prevents recognition of those epitopes by specific antibodies [13]. Beneficial effect of TG was also observed on immunoreactivity modification of cereals proteins. In this way, it can be used to influence the clinical manifestation of food sensitivity. Watanabe et al. [14] showed that the use of TG allows to obtain hypoallergenic flour from wheat, which can be consumed by persons with hypersensitivity to wheat. In light of the above, diverse roles of TGs in immune responses are very intriguing. Poland's national data indicated that cross-linking by TG caused decrease of gluten immunoreactivity [15], which raises hopes for TG use to modify nutrition of CD/DH patients. Thus, having knowledge of TGs is essential for understanding the pathogenesis of CD and DH [16], in which the production of autoantibodies to TGs (as a result of chain of events initiated by deamidation of glutamine residue in gliadin catalyzed by tTG) might surprisingly be of minor significance compared with benefits resulting from TGs-mediated cross-linking of proteins. Regardless of pathogenetical considerations, direct immunofluorescence test (DIF) of nonlesional skin remains definitive laboratory test for diagnosing DH [7]. However, due to numerous clinical manifestations of GSE (including DH), the use of serological techniques becomes helpful in clinical practice lowering the need for performing invasive gut biopsies [17]. 

Receiver operating characteristic (ROC) curves represent a relationship between sensitivity and specificity of a laboratory test over all possible diagnostic cutoff values. So, using ROC curves in laboratory medicine should be a common practice to facilitate clinical decision making.

## 2. Aim of the Study

The aim of this study was to investigate the diagnostic accuracy of ELISA tests evaluating serum IgA antibodies to tTG and npG in Slavic DH patients through performing the receiver operating characteristic (ROC) and determining proper cutoff values. Also, the aim was to assess correlations between levels of anti-tTG and anti-npG IgA antibodies in IgA/neutrophil-mediated DH and non-DH patients and healthy individuals. 

## 3. Material and Methods

Altogether, sera from 80 Slavic individuals were tested. Serum samples were obtained from patients with DH (19 men and 12 women) with skin rash active enough to prompt them to seek dermatological attention and IgA/neutrophil-mediated non-DH dermatoses (25 patients as positive control involved 17 cases of linear IgA bullous dermatosis, 4 cases of IgA pemphigus, 3 cases of epidermolysis bullosa acquisita and 1 case of subcorneal pustular dermatosis) as well as from healthy individuals (negative control, 24 donors). The DH group and IgA/neutrophil-mediated non-DH dermatoses group served as mutual control groups. The diagnosis of DH in all cases suspected of having DH at the clinical level was established when cutaneous IgA deposition in any of seven possible diagnostic patterns was seen with conventional DIF and corroborated by histological picture with hematoxylin and eosin (H + E) staining. To distinguish from DH and establish the clinical diagnoses of IgA/neutrophil-mediated non-DH dermatoses, DIF and H + E staining were also done. Testing of sera with biochemical molecular techniques was done in cases requiring broader differential diagnostics. DH serum samples were collected during the period from February 2010 through July 2011, and all serum samples were evaluated in the Cutaneous Histopathology and Immunopathology Section, Department of Dermatology, Poznan University of Medical Sciences, Poland. All healthy controls were not relatives of DH patients and gave no history of intolerance to gluten. 

The levels of serum IgA autoantibodies against the fusion protein containing nonapeptides of gliadin were evaluated with Antigliadin (GAF-3X) ELISA (Euroimmun, Germany) with the manufacturer's cutoff value 25 RU/mL. The levels of serum IgA autoantibodies against tTG were assessed with Anti-tTG ELISA (Euroimmun, Germany) with the manufacturer's cutoff value 20 RU/mL. Both tests are recommended by the producer as useful in DH diagnosis. All measurements were made using a programmable ELISA reader with MikroWin 2000 software by a single operator following the manufacturer's instructions. All tests were performed with meticulous care to eliminate the risk of intralaboratory errors.

Antigens which were used in the tests were obtained with molecular engineering methods. The antigen in Antigliadin (GAF-3X) ELISA is a recombinant fusion protein, which was created using DNA sequences encoding gliadin-analogous fusion peptide, GAF. These gliadin-analogous peptides consist of three repeated sequences to increase the diagnostic efficacy. The created protein composition involves synthetic nonapeptide which is gliadin analoque and nonapeptide of digested gliadin which was deamidated by transglutaminases. Antigen in Anti-tTG ELISA is recombinant tTG. The expression of proper human cDNA was done with baculovirus vector in insect cell systems.

In this study, the ROC analysis was used for determining cutoff points for the best sensitivity and specificity of tTG/npG ELISA tests and evaluating their ability to discriminate disease-affected from normal cases. Statistical analysis was performed by use of MedCalc statistical software (http://www.medcalc.org/). Kruskal-Wallis test with post hoc Dunn's test was performed to detect significant differences between three studied groups. Spearman's rank correlation coefficient was computed to find out associations between levels of IgA antibodies to tTG and npG in all groups. 

## 4. Results

**Figure 2:**
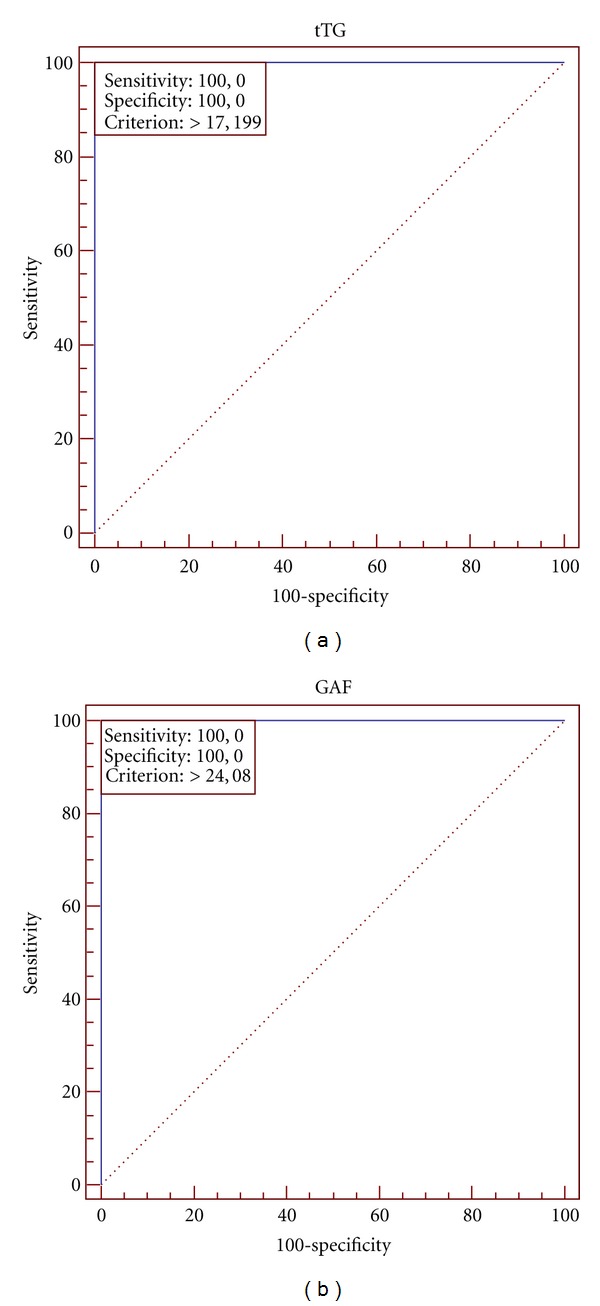
ROC analysis for DH patients and healthy controls. (a) shows the plot for Anti-tTG IgA ELISA, and (b) shows Antigliadin (GAF-3X) IgA ELISA. The cutoff point marked as criterion (17.199 and 24.08, resp.).

**Figure 3:**
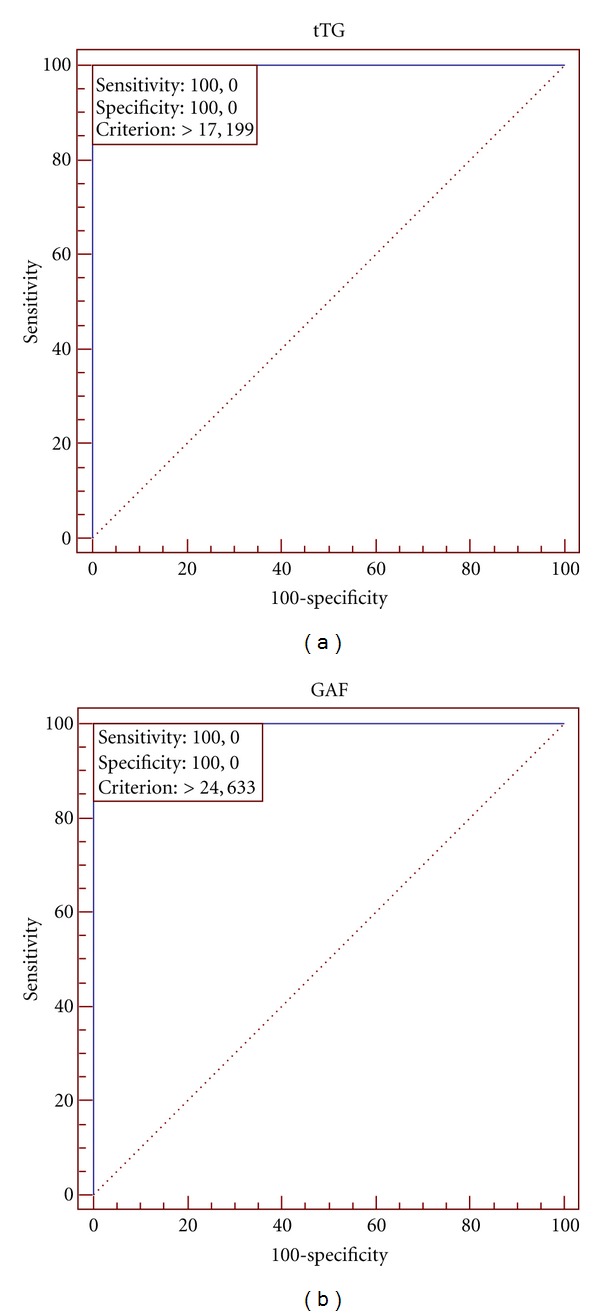
ROC analysis for all examined groups. (a) shows the plot for Anti-tTG IgA ELISA, and (b) shows Antigliadin (GAF-3X) IgA ELISA.The cutoff point marked as criterion (17.199 and 24.633, resp.).

**Figure 4:**
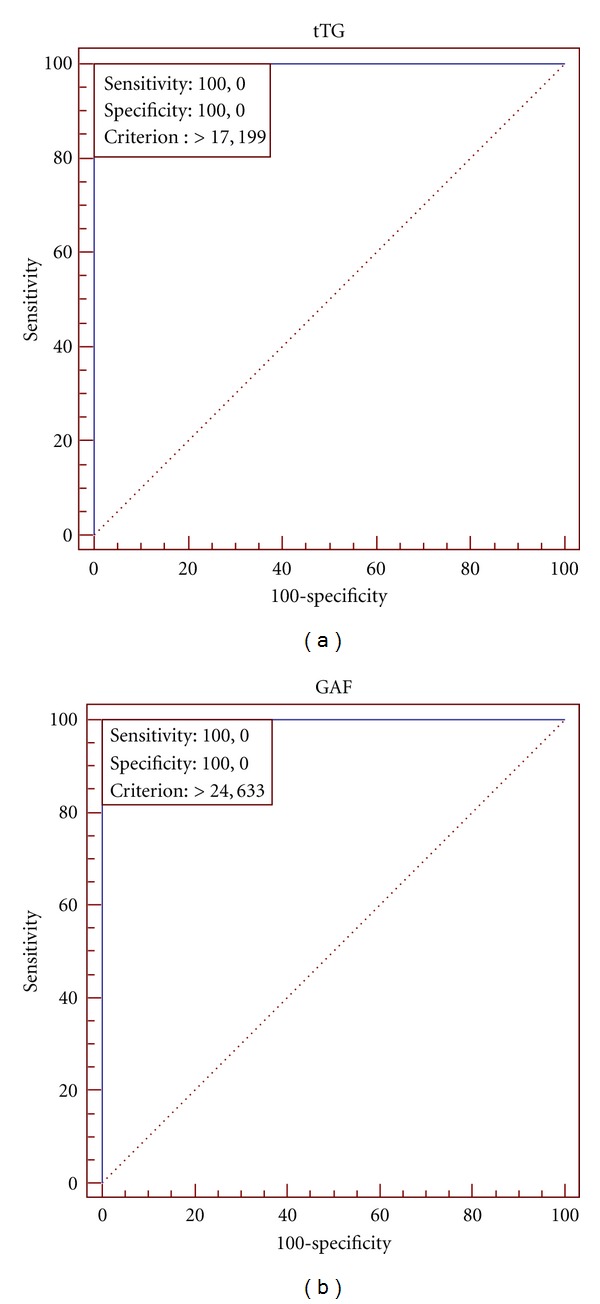
ROC analysis for DH patients and IgA/neutrophil-mediated non-DH dermatoses group. (a) shows the plot for Anti-tTG IgA ELISA, and (b) shows Antigliadin (GAF-3X) IgA ELISA. The cutoff point marked as criterion (17.199 and 24.633, resp.).

Results of statistical analysis of three groups of examined patients are presented in Table 1. The ROC plots created for various combinations of examined groups are shown in Figures 2, 3, and 4. 

The optimal cut-off for the Anti-tTG IgA ELISA in all combinations (DH patients versus healthy control; DH patients versus IgA/neutrophil-mediated non-DH dermatoses; combined groups) of examined groups is 17.199 RU/mL (manufacturer's cut-off is 20 RU/mL). The optimal cut-off for the Antigliadin (GAF-3X) IgA ELISA is 24.633 RU/mL in DH patients versus IgA/neutrophil-mediated non-DH dermatoses and in combined groups (manufacturer's cut-off is 25 RU/mL). The optimal cut-off for the Antigliadin (GAF-3X) IgA ELISA is 24.08 RU/mL in DH patients versus healthy controls (manufacturer's cut-off is 25 RU/mL). It is noteworthy that in each combination the area under the ROC curve (AUC) is 1.00.

Regarding values of both anti-tTG and anti-npG IgA, differences between healthy control and DH patients (*P* < 0.001) were significant as well as between IgA/neutrophil-mediated non-DH dermatoses and DH patients (*P* < 0.001), whereas differences between healthy control and IgA/neutrophil-mediated non-DH dermatoses were not revealed (*P* > 0.05). The analysis of correlation showed statistically significant correlations between levels of IgA antibodies to tTG and npG in both DH group (*r* = 0.4149) and the group of IgA/neutrophil-mediated non-DH dermatoses (*r* = 0.7890), whereas there was no such correlation in healthy controls (*r* = 0.2231).

The following positive results were obtained in examined groups: (i) in case of anti-tTG IgA: 90% of DH patients, 4% of IgA/neutrophil-mediated non-DH dermatoses patients, and 0% in healthy controls; (ii) in case of anti-npG IgA: 90% of DH patients, 8% of IgA/neutrophil-mediated non-DH dermatoses patients, and 0% in healthy controls. 

## 5. Discussion

In our Slavic DH patients, the ratio between men and women was 1.6 : 1, which is consistent with the reports that DH affects men slightly more often [18, 19].

In this study, we attempt to assess diagnostic usefulness and accuracy of tTG/npG ELISA tests in the management of Slavic DH patients. Curiously, the research literature data about DH and studies on its pathomechanism are amazingly scanty in relation to the other autoimmune blistering dermatoses, while it seems that DH pathogenesis is far more complex. As mentioned above, GSE, both CD and DH, is a growing medical problem, particularly among young people, with nutritional impact on their health status. Thus, specific, reliable, and objective criteria for diagnosing and monitoring of DH should be established. DIF of nonlesional skin remains a definitive laboratory test for diagnosing this disease, however its invasiveness is a serious limitation for screening. Indirect immunofluorescence is a time-consuming, expensive, and subjective technique; then, ELISA seems to be free from those disadvantages. At present, ELISA is the method of choice for serological screening of DH. However, there is a problem what kind of ELISA-based test is the best. The medical diagnostics market offers a wide range of ELISA kits with biotechnologically obtained eTG, tTG, and npG as the most frequent antigen sources for diagnosing DH. Literature data indicated that there are discrepancies regarding the specificity and repeatability of test results. Some findings showed that 52% of DH patients have anti-eTG IgA elevated [20], which is consistent with our previous study [21]. On the other hand, several communications revealed that more than 90% of DH patients have anti-eTG IgA elevated [19, 22]. Such divergent results might be caused by evaluating series of DH patients differing in severity of cutaneous rash. In the light of this, there is an urgent need to find/develop a new or modified antigen/epitope that would make the DH diagnostics more accurate. Thus, the usefulness of tTG/npG ELISA-based tests should be considered. Literature associated with this problem demonstrated that there is also incompatibility about the level of anti-tTG IgA. Some investigators [20] obtained only 25% of anti-tTG IgA-positive results in DH patients, whereas other researchers achieved about 79% of positive results in their patients [19, 23]. Our own experience in this area is satisfactory: 90% of DH patients examined in this study had anti-tTG IgA above normal range. Important issue remains the composition and structural design of the antigen/epitope. Byrne et al. [24] indicated that the use of novel mutagenic variant of tTG lacking the catalytic triad decreases the binding of IgA to the mutant tTG with the mean reduction of 58% in DH and even bigger mean reduction of 79% in CD samples. Fernández et al. [25] analyzed six different human anti-tTG ELISA kits. This group of researchers showed that there are differences in the sensitivity and specificity of the human tTG ELISA assays. Furthermore, they suggested that diagnostic accuracy of tests was significantly improved by adjusting the cutoff thresholds according to ROC curve analyses, which was done in our study. Manufacturer's cut-off is not standardized to each laboratory conditions, therefore the standardization based on ROC curve analyses should be recommended to all ELISA tests performed for diagnostic purposes. Interestingly, Fernández et al. [25] demonstrated that the correction of the cut-off with the use of the ROC curve analysis modifies the decision limit in more than 50% in five of the six examined anti-human tTG ELISA kits. Hence, there is the evidence that the way/source of production and further modification of antigen have the impact on test accuracy. Researchers and diagnosticians should take it into account before the choice of the appropriate test.

Recently [26, 27], investigators indicated the usefulness of synthetic deamidated gliadin-derived peptides (GDR) as antigen, which is useful for the detection of sensitivity to gluten in anti-tTG IgA seronegative DH patients. There is a hypothesis that they are the most reliable tools for identification of gluten sensitivity in DH patients [12]. Our study demonstrated the presence of anti-npG IgA in 90% of examined DH patients, which may imply that usage of the ELISA test measuring IgA antibodies to this antigen broadens the information necessary to make the correct diagnosis. Presented results correspond to those obtained by Kasperkiewicz et al. [23] indicating that 84% of DH patients have IgA to npG detected with GAF-3X ELISA. In this study, we determined the cutoff value for examined DH patients in anti-tTG ELISA as the level 17.199 RU/mL (for all examined groups), while the manufacturer's cut-off is 20 RU/mL. In anti-npG ELISA, the cut-off for examined population is 24.633 RU/mL (for 2 combinations of groups) and 24.08 RU/mL (for DH and healthy groups), while the manufacturer's cut-off is 25 RU/mL. Thus, there are negligible differences between them (especially in case of antigliadin GAF-3X ELISA). It can be explained by the fact that kits used are well standardized in a genetically similar population. It is known that the cutoff point can vary depending on examined population, for example, different cutoff value of anti-tTG ELISA for Italian and Spanish populations [28, 29]. Interestingly, the cutoff points in our studied groups were almost identical. It may be due to the fact that diseased group, IgA/neutrophil-mediated non-DH dermatoses, was chosen as pathogenetically most closely related to DH, as far as cutaneous pathology is concerned. Moreover, AUC obtained in this study was equal 1, which may be due to a perfect separation of the values of the examined groups. It should be stressed here that we had precise inclusion criteria and each diagnosis had to be confirmed with the combination of microscopic and biochemical/molecular techniques. Thus, using the new cutoff values derived from ROC curves, the diagnostic accuracy of the tests is improved (sensitivity and specificity of 100%). It could be due to fact that the tests were adapted to our native population. 

There were no individuals with positive anti-tTG/npG IgA antibodies in the healthy controls, although we did not make the biopsy for DIF in order to rule out DH in these subjects presenting no cutaneous lesions whatsoever. In addition, the percentage of cases with positive anti-tTG/npG IgA antibodies in IgA/neutrophil-mediated non-DH dermatoses group has been quite low (4% of anti-tTG ELISA and 8% of anti-npG ELISA). Still, it cannot be excluded that some such cases had low-grade GST with no overt clinical symptoms in addition to their IgA/neutrophil-mediated non-DH dermatosis. Interestingly, findings obtained by Ludvigsson et al. [30] in a nationwide cohort study indicated that individuals with CD were at increased risk of psoriasis both before and after CD diagnosis. Thus, one should be aware that CD can coexist with other dermatoses, not only with DH. In the light of the above, it is suggested that recognition of anti-npG IgA alone does not mean that one is dealing with DH as a cutaneous manifestation of GSE, and DIF of the uninvolved skin still remains crucial for diagnosing DH.

In our study, we demonstrated that the determination of anti-tTG/npG IgA by means of ELISA is a precise method to broaden the body of knowledge about DH patients in Slavic population. However, what was noticed by Fernández et al. [25] is that it is necessary to select the ELISA kit with the highest sensitivity and specificity and recalculate the cutoff threshold using samples from any given native population. This sequence of actions is essential for the laboratory diagnostician to provide reliable information to the clinician to facilitate making right decision on the implementation of troublesome therapy with the risk of potentially dangerous sideeffects. 

Statistically significant correlation between levels of IgA antibodies to tTG and npG in DH group (*r* = 0.4149) found in this study and no such correlation in healthy controls (*r* = 0.2231) might suggest that the anti-tTG and anti-npG IgA antibodies in the IgA/neutrophil-mediated DH, but not in healthy individuals, are produced in the coordinated way in contrast to healthy individuals. These findings are in agreement with our previous data [31]. This might correspond with a suggestion that, as the catalytic site of tTG seems to be targeted by IgA autoantibodies, intermolecular epitope spreading from the gliadin epitopes to the catalytic site of tTG does take place in CD [24]. 

In recent years, the cutaneous immunopathology of DH has become the area of extensive studies in humans and using animal models of the disease [21, 32, 33]. We feel that the key issue in understanding the blister formation in DH is not simply linking it to the GSE, but evaluating instead local cutaneous factors, conceivably neutrophils' Fc receptors involvement, at the human skin level.

Nonetheless, it should be kept in mind, as far as the issue of importance of npG in pathogenesis and diagnostics of GSE is concerned, that within the gliadin peptide the N-terminal proline residue and the C-terminal glutamine residues were reported to be essential for antibody recognition in addition to the deamidated glutamine residue [34]. Finally, our data might suggest that Anti-tTG IgA ELISA is marginally superior to Antigliadin (GAF-3X) IgA ELISA for differential diagnosis of cutaneous itchy rashes suspected to be DH at the clinical level. Plausibly, in connection with the data that IgA1 deposits predominate in the skin of DH sufferers, modification of the above ELISA tests to enable the determination of IgA1 subclass antibodies to tTG and npG would be even more valuable for differentiating IgA/neutrophil-mediated dermatoses.

## 6. Conclusions

IgA antibodies to tTG and npG are detectable in the vast majority of DH Slavic patients. Tests based on these antigens tailored to native/local populations, for example, Slavic assessed in this study, broaden DH diagnostics, being useful for the detection of gluten sensitivity in DH. However, DIF of the uninvolved skin still remains the reference method for diagnosing DH. 

It seems that the anti-tTG and anti-npG IgA antibodies in the IgA/neutrophil-mediated DH, but not in healthy individuals, are produced in the coordinated way implying their causal relationship. 

## Figures and Tables

**Table 1:** Statistical analysis of the three groups of Slavic subjects studied.

IgA antibodies evaluated with	Examined group	Number of patients	Mean	SD	Dunn's test
Anti- tTG ELISA (RU/mL)	Group I	24	2.35	1.58	Group I versus group III *P* < 0.001 Group II versus group III *P* < 0.001 Group I versus group II *P* > 0.05
	Group II	25	13.57	46.57	
	Group III	31	429.20	435.23	
Antigliadin (GAF-3X) ELISA (RU/mL)	Group I	24	1.43	3.32	Group I versus group III *P* < 0.001 Group II versus group III *P* < 0.001 Group I versus group II *P* > 0.05
	Group II	25	15.10	44.17	
	Group III	30	271.00	499.25	

Explanations: group I: healthy controls, group II: IgA/neutrophil-mediated non-DH dermatoses group, group III: DH patients. SD: standard deviation.

## References

[1] Dmochowski M (2006). Dermatitis herpetiformis. *Autoimmune Blistering Dermatoses*.

[2] Duś M, Dańczak-Pazdrowska A, Gornowicz J, Bowszyc-Dmochowska M, Dmochowski M (2009). Transient manifestation of dermatitis herpetiformis in a female with familial predisposition induced by propafenone. *Advances in Dermatology and Allergology*.

[3] Zone JJ Skin manifestations of celiac disease.

[4] Reunala TL (2001). Dermatitis herpetiformis. *Clinics in Dermatology*.

[5] Olbricht SM, Flotte TJ, Collins B, Chapman CM, Harrist TJ (1986). Dermatitis herpetiformis. Cutaneous deposition of polyclonal IgA1. *Archives of Dermatology*.

[6] Wojnarowska F, Delacroix D, Gengoux P (1988). Cutaneous IgA subclasses in dermatitis herpetiformis and linear IgA disease. *Journal of Cutaneous Pathology*.

[7] Gornowicz J, Bowszyc-Dmochowska M, Dmochowski M (2010). IgA antibodies against fusion protein containing modified nonapeptide of gliadin in dermatitis herpetiformis. *Dermatologia Kliniczna*.

[8] Sárdy M, Kárpáti S, Merkl B, Paulsson M, Smyth N (2002). Epidermal transglutaminase (TGase 3) is the autoantigen of dermatitis herpetiformis. *Journal of Experimental Medicine*.

[9] Dieterich W, Laag E, Bruckner-Tuderman L (1999). Antibodies to tissue transglutaminase as serologic markers in patients with dermatitis herpetiformis. *Journal of Investigative Dermatology*.

[10] Gornowicz-Porowska J, Bowszyc-Dmochowska M, Dmochowski M (2011). Autoimmunity-driven enzymatic remodeling of the dermal-epidermal junction in bullous pemphigoid and dermatitis herpetiformis. *Autoimmunity*.

[11] Schwertz E, Kahlenberg F, Sack U (2004). Serologic assay based on gliadin-related nonapeptides as a highly sensitive and specific diagnostic aid in celiac disease. *Clinical Chemistry*.

[12] Sugai E, Smecuol E, Niveloni S (2006). Celiac disease serology in dermatitis herpetiformis. Which is the best option for detecting gluten sensitivity?. *Acta Gastroenterologica Latinoamericana*.

[13] Wróblewska B, Kaliszewska A, Malinowska E, Troszyńska A (2011). Immunoreactivity of transglutaminase cross-linked milk proteins in fermentem milk product obtained with Lactobacillus acidophilus. *Advances in Dermatology and Allergology*.

[14] Watanabe M, Miyakawa J, Ikezawa Z, Suzuki Y, Hirao T (2004). Production of hypoallergenic rice by enzymatic decomposition of constituent proteins. *Journal of Food Science*.

[15] Leszczyńska J (2009). Immunoreaktywność wybranych składników żywności i sposoby jej zmniejszania. (The methods of reducing immunoreactivity of the food allergens). *Scientific Notebooks, Dissertations/Technical University of Lodz*.

[16] Lorand L, Graham RM (2003). Transglutaminases: crosslinking enzymes with pleiotropic functions. *Nature Reviews Molecular Cell Biology*.

[17] Zintzaras E, Germenis AE (2006). Performance of antibodies against tissue transglutaminase for the diagnosis of celiac disease: meta-analysis. *Clinical and Vaccine Immunology*.

[18] Braun-Falco O, Plewig HH, Giliński W, Wolska H, Zaborowski P (2002). Dermatitis herpetiformis. *Dermatology T I*.

[19] Rose C, Bröcker EB, Zillikens D (2010). Clinical, histological and immunpathological findings in 32 patients with dermatitis herpetiformis Duhring. *JDDG*.

[20] Hull CM, Liddle M, Hansen N (2008). Elevation of IgA anti-epidermal transglutaminase antibodies in dermatitis herpetiformis. *British Journal of Dermatology*.

[21] Bowszyc-Dmochowska M, Seraszek A, Kaczmarek E, Gornowicz J, Dmochowski M (2009). Low strength of correlation between the level of serum IgA antibodies to epidermal transglutaminase and the intensity of neutrophil elastase expression in lesional skin in dermatitis herpetiformis. *The Open Autoimmunity Journal*.

[22] Rose C, Armbruster FP, Ruppert J, Igl BW, Zillikens D, Shimanovich I (2009). Autoantibodies against epidermal transglutaminase are a sensitive diagnostic marker in patients with dermatitis herpetiformis on a normal or gluten-free diet. *Journal of the American Academy of Dermatology*.

[23] Kasperkiewicz M, Dähnrich C, Probst C (2011). Novel assay for detecting celiac disease-associated autoantibodies in dermatitis herpetiformis using deamidated gliadin-analogous fusion peptides. *Journal of the American Academy of Dermatology*.

[24] Byrne G, Ryan F, Jackson J, Feighery C, Kelly J (2007). Mutagenesis of the catalytic triad of tissue transglutaminase abrogates coeliac disease serum IgA autoantibody binding. *Gut*.

[25] Fernández E, Riestra S, Rodrigo L (2005). Comparison of six human anti-transglutaminase ELISA-tests in the diagnosis of celiac disease in the Saharawi population. *World Journal of Gastroenterology*.

[26] Sugai E, Hwang HJ, Vázquez H (2010). New serology assays can detect gluten sensitivity among enteropathy patients seronegative for anti-tissue transglutaminase. *Clinical Chemistry*.

[27] Jaskowski TD, Donaldson MR, Hull CM (2010). Novel screening assay performance in pediatric celiac disease and adult dermatitis herpetiformis. *Journal of Pediatric Gastroenterology and Nutrition*.

[28] Martini S, Mengozzi G, Aimo G, Pagni R, Sategna-Guidetti C (2001). Diagnostic accuracies for Celiac disease of four tissue transglutaminase autoantibody tests using human antigen. *Clinical Chemistry*.

[29] Vivas S, Ruiz de Morales JM, Martínez J (2003). Human recombinant anti-transglutaminase antibody testing is useful in the diagnosis of silent coeliac disease in a selected group of at-risk patients. *European Journal of Gastroenterology and Hepatology*.

[30] Ludvigsson JF, Lindelöf B, Zingone F, Ciamci C (2011). Psoriasis in nationwide cohort study of patients with celiac disease. *Journal of Investigative Dermatology*.

[31] Dmochowski M, Gornowicz J, Seraszek A (2011). Cutaneous expression of neutrophil elastase in relation to IgA autoantibodies to nonapeptides of gliadin and tissue transglutaminase in dermatitis herpetiformis. *Journal of Investigative Dermatology*.

[32] Mihai S, Sitaru C (2007). Immunopathology and molecular diagnosis of autoimmune bullous diseases. *Journal of Cellular and Molecular Medicine*.

[33] Zone JJ, Schmidt LA, Taylor TB (2011). Dermatitis herpetiformis sera or goat anti-transglutaminase-3 transferred to human skin-grafted mice mimics dermatitis herpetiformis immunopathology. *Journal of Immunology*.

[34] Petersen NH, Hansen PN, Houen G (2011). Fast and efficient characterization of an anti-gliadin monoclonal antibody epitope related to celiac disease using resin-bound peptides. *Journal of Immunological Methods*.

